# Fabricated High-Strength, Low-Elastic Modulus Biomedical Ti-24Nb-4Zr-8Sn Alloy via Powder Metallurgy

**DOI:** 10.3390/ma16103845

**Published:** 2023-05-19

**Authors:** Amy X. Y. Guo, Bin Cao, Zihan Wang, Xiao Ma, Shan Cecilia Cao

**Affiliations:** 1 Materials Genome Institute, Shanghai University, Shanghai 200444, China; guoxiaoyun@shu.edu.cn (A.X.Y.G.); bcao@shu.edu.cn (B.C.);; 2 Institute for the Conservation of Cultural Heritage, Shanghai University, Shanghai 200444, China

**Keywords:** Ti2448 alloy, microstructure analysis, porosity, mechanical properties

## Abstract

With the huge demands of an aging society, it is urgent to develop a new generation of non-toxic titanium alloy to match the modulus of human bone. Here, we prepared bulk Ti2448 alloys by powder metallurgy technology, and focused on the influence of the sintering process on the porosity, phase composition, and mechanical properties of the initial sintered samples. Furthermore, we performed solution treatment on the samples under different sintering parameters to further adjust the microstructure and phase composition, so as to achieve strength enhancement and reduction of Young’s modulus. Solution treatment can effectively inhibit the continuous α phase precipitated along the grain boundaries of the β matrix, which is beneficial to the fracture resistance. Therefore, the water-quenched sample exhibits good mechanical properties due to the absence of acicular α-phase. Samples sintered at 1400 °C and subsequently water quenched have excellent comprehensive mechanical properties, which benefit from high porosity and the smaller feature size of microstructure. To be specific, the compressive yield stress is 1100 MPa, the strain at fracture is 17.5%, and the Young’s modulus is 44 GPa, which are more applicable to orthopedic implants. Finally, the relatively mature sintering and solution treatment process parameters were screened out for reference in actual production.

## 1. Introduction

Titanium alloy is widely used as implant material because of its high specific strength, low elastic modulus, good corrosion resistance, and excellent biocompatibility, and titanium alloy has become an important biomedical material [1,2,3,4]. Ti-6Al-4V is the most widely used titanium alloy in clinical medicine that has been thoroughly studied, including manufacturing techniques, microstructure characterization, mechanical performance, and corrosion resistance [5,6,7]. However, there is still an urgent need to further develop new type titanium alloys. First, the elastic modulus of titanium alloy implants (~110 GPa) is much higher than that of human bone (12–30 GPa) [8,9]. Orthopedic implants with incompatible elastic modulus could generate stress shielding after implantation, leading to loosening or fracture of the implant in the human body [10,11]. Second, alloying elements such as Ni, Al, and V are toxic and allergenic, and they may result in Alzheimer’s and neurological diseases when they are released into the human body through corrosion [12,13].

In order to overcome the above problems, a new generation of non-toxic medical titanium alloys with lower modulus and better biocompatibility have been developed in recent years [14,15]. Bordbar-Khiabani et al. [16] developed Ti-Nb-Zr-Si alloy, which exhibit superior corrosion resistance and favorable cell-materials interactions in vitro. Hao et al. devised the Ti-24Nb-4Zr-8Sn (wt.%) alloy, which is known as the representative of the next generation of medical materials because of its excellent mechanical properties and biocompatibility [17,18]. First of all, polycrystalline Ti2448 has high strength (~1150 MPa), low elastic modulus (~56 GPa), large recoverable strain (~3.3%), and a weak grain refinement strengthening effect [17]. Especially the <100> oriented single crystal exhibits weak nonlinear elasticity with large elastic strain (~2.5%) and low Young’s modulus (27.1 GPa) [19]. Moreover, Ti2448 alloy does not contain Al and V elements, which are toxic to the human body. Nune et al. reported that the alloy formed fine apatite-like crystals in simulated body fluids, which confirmed the excellent biological activity of the alloy [20]. Obbard et al. [21] found that oxygen reduces the non-line recoverable strain, shortens the stress plateau and obscures the double yield points of forged Ti2448 alloy. Li et al. [21] investigated the tensile deformation mechanism of a high-oxygen Ti2448 alloy fabricated by powder metallurgy, and they pointed out that both dislocation slide and stress-induced phase transformation contribute to the plastic deformation of the alloy. Tang et al. [22] designed experiments to explore the role of 3D printed Ti2448 in macrophage activation and related osteogenesis and angiogenesis. More research focused on the biocompatibility of Ti2448 alloys prepared by powder metallurgy [23,24,25].

Furthermore, research of the ball milling procedure has greatly promoted the development of Ti-based powder metallurgy technology [26,27]. Yim et al. [28] found that mechanical stimulation was able to suppress the smoke of gas-atomized Ti-48Al-2Cr-2Nb powder using Al_2_O_3_ and WC ball milling. Salur et al. [29] studied the effect of ball milling time on the structural characteristics and mechanical properties of the nano-sized Y_2_O_3_ particle, which reinforced aluminum matrix composites produced by the powder metallurgy route. Pradeep et al. [30] synthesized the Ti-Mg-Sr alloy powder by process of mechanical alloying, and the grain size of the Ti-Mg-Sr particles decreased to a nanoscale regime from a micron scale. The developed novel Ti-Mg-Sr alloy could be useful for biomedical application.

However, optimizing the preparation protocol to meet the needs of the medical implant market is far-reaching and critical. Common methods include powder metallurgy [31], electron beam melting [2], and selective laser melting [32]. Among them, although powder metallurgy is relatively traditional, it has economic advantages. As a near-net shape technology, it can reduce machining processes and improve the production efficiency of structural parts [33]. The application of powder metallurgy technology can further popularize the practical application fields of titanium alloy materials, especially in civil fields [34]. Here we prepared bulk Ti2448 alloys by powder metallurgy technology, and focused on the influence of the sintering process on the porosity, phase composition, and mechanical properties of the initial sintered state. Furthermore, we performed solution treatment on the samples under different sintering parameters to further tunning of the microstructure and phase composition, so as to achieve strength enhancement and reduction of Young’s modulus. Finally, the relatively mature sintering and solution treatment process parameters were screened out to promote the wide application of Ti2448 in medical implants.

## 2. Methods and Materials

### 2.1. Materials and Processing

**Figure 1:**
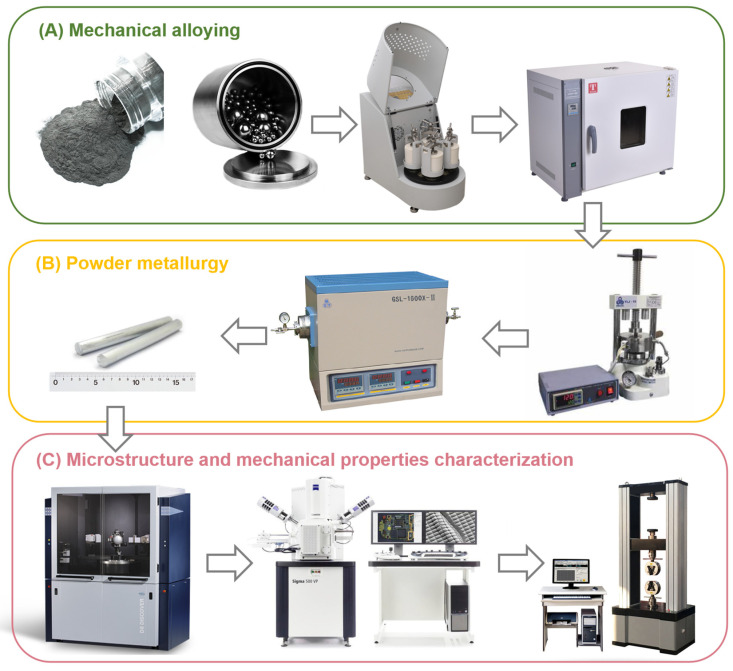
A schematic representation of the main stages of experimental work performed in the present study: (**A**) mechanical alloying procedure; (**B**) powder metallurgy procedure; (**C**) equipment used for microstructure and mechanical properties characterization.

A schematic representation of the main stages of experimental work performed in the present study are shown in Figure 1. Ti2448 alloy was manufactured by using elemental powders, which are more flexible and for which it is easier to adjust the composition. High-purity Ti, Nb, Zr, and Sn powders as raw material, and we mixed the powders at a mass ratio of 64:24:4:8 and then put them into a ball miller (QM-3SP4, Changsha Miqi Instrument Equipment Co., Ltd., Changsha, China) to achieve mechanical alloying. We adopted ethanol as a process control agent (PCA) to avoid cold welding and bonding between the powder particles and the balls as well as the agglomeration of the powder during milling. The grinding had a speed of 300 rpm and lasted for 20 h. After ball milling, the slurry was dried at 60 °C for 10 h under vacuum, and the powder and grinding balls were separated using a 300-mesh sieve to obtain pre-alloyed powder. Subsequent cold pressing (YLJ-CSP-15, MTI Corporation, Hefei, China) was used to fabricate green compacts (Φ10 mm × 15 mm).

The green compacts were sintered in a Al_2_O_3_ tube furnace under a flowing Ar atmosphere, during which the samples were sintered at different temperatures (1400 °C, 1450 °C and 1500 °C) for 12 h and then cooled down to room temperature with the furnace. The as-sintered samples were solution-treated at 1000 °C for 1 h and subsequently water quenched.

### 2.2. Microstructure Characterization

The X ray diffraction (XRD) measurements were carried out with a Bruker D8 advance (D8, Bruker, Germany), with a Cu target operating at the anode target voltage of 40 kV, where the target current is 30 mA, the scanning speed is 0.01°/s, the integration time of one step is 2 s, and the scanning angle is 30°~90°. Whole Pattern fitting of powder X-ray diffraction was by Expectation Maximum (WPEM) software (WPEM, Beta version). The primary goal of WPEM is to develop an open-source diffraction whole pattern fitting software that offers high solution efficiency and precision. WPEM also provide the open-source address (github.com, accessed on 30 March 2023/Bin-Cao/WPEM), allowing researchers and scientists to access and utilize this powerful software for their structural analysis needs. The WPEM analyzes a whole XRD patten with the Bragg law-based XRD theory and the Expectation-Maximum algorithm. The software is particularly effective in determining complex crystal structures and decoupling heavily overlapped Bragg peaks in the whole XRD pattern.

The microstructure and component distribution were characterized by the scanning electron microscopy (SEM, Sigma 500, Carl Zeiss, Jena, Germany) with an operating voltage of 15 kV and a working distance of 8.0 mm. The samples for microstructure observation were grinded and polished, and were then etched using a mixed solution of HF, HNO_3_, and H_2_O with a volume ratio of 1:3:7. The compositions were assessed by energy dispersive spectroscopy (EDS, AZTEC, Oxford Instrument, Abingdon, UK) analysis.

### 2.3. Mechanical Testing

Specimens for compressive tests were cut from the rods using an electro-discharge machine. The compressive tests were conducted at room temperature using a universal testing machine (Material Testing System Model 810, Eden Prairie, MN, USA) equipped with an extensometer and the strain rate was set at 1.0 × 10^−3^ s^−1^. The mechanical response under the compressive load condition was investigated by plotting the load vs. displacement curve until the samples get fractured. The Young’s modulus was determined from the slope of the stress vs. strain curve for each sample.

## 3. Results and Discussion

### 3.1. Powder Size and Microstructure Characterization

**Figure 2:**
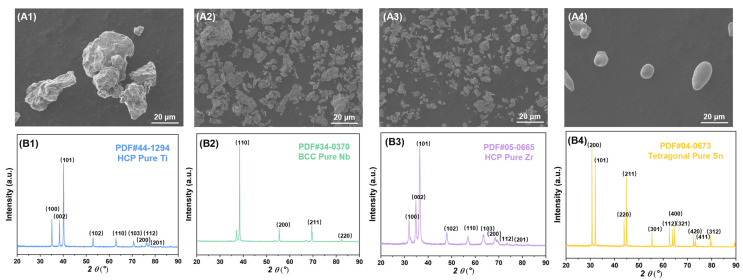
SEM images and XRD spectrums of raw powder: (**A1**,**B1**) titanium powder; (**A2**,**B2**) niobium powder; (**A3**,**B3**) zirconium powder; (**A4**,**B4**) tin powder.

**Figure 3:**
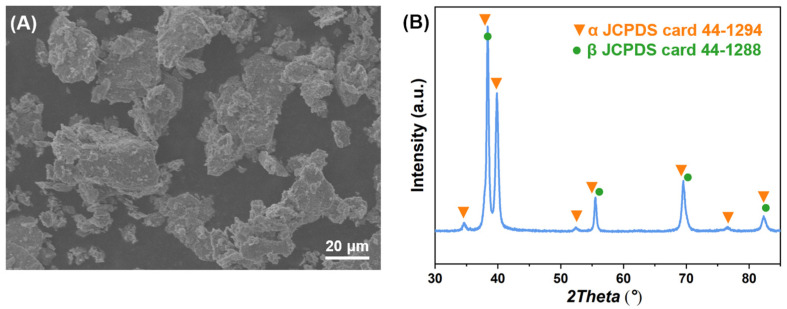
(**A**) SEM images and (**B**) XRD spectrums of pre-alloyed powder.

The SEM images of the raw powders are shown in Figure 2. It can be obtained from the statistical results of multiple images that the non-spherical titanium powder has a diameter of 30 μm, the non-spherical niobium powder has a diameter of 17 μm, the non-spherical zirconium powder has a diameter of 10 μm, the spherical tin powder has a diameter of 10 μm. Their crystal structures correspond to hexagonal close packet (HCP), body centered cubic (BCC), HCP and tetragonal system, respectively. The Ti2448 powder after ball milling has a loose layered structure and has undergone mechanical alloying, as shown in Figure 3, with a diameter of about 15 μm and composed of α (HCP) and β (BCC) phases. This also shows that ball milling makes the elemental powder fully and uniformly mixed, which is helpful for the subsequent sintering process.

### 3.2. Microstructure Characterization of As-Sintered Samples

**Figure 4:**
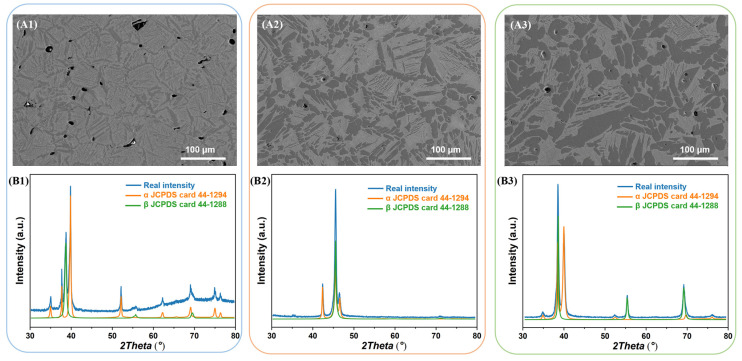
SEM images and XRD spectrums of as-sintered samples: (**A1**,**B1**) S1400 sample; (**A2**,**B2**) S1450 sample; (**A3**,**B3**) S1500 sample.

Figure 4 shows the microstructure and XRD patterns of the green samples, which are sintered at different temperatures, 1400 °C, 1450 °C, and 1500 °C, respectively. We abbreviate them as S1400, S1450, and S1500, respectively. First of all, the microstructure of the samples belong to the transformation β structure category, which is derived from sintering temperatures higher than the β transition temperature (850 °C) [35] and the sintered samples cooled slowly with the furnace. The mixed structure formed by the decomposition of the β phase, and usually consisting of alternating lamellar α and β arrange composition. The secondary α phase inside the grains, though, while the continuous α phase is precipitated along the grain boundaries of the β matrix, which is called the grain boundary α-phase (GB-α). The GB-α in titanium alloys is usually formed in conventionally manufactured titanium alloys due to the slow cooling process [36,37,38,39]. However, the characteristic size and porosity of the samples prepared under the three sintering processes are completely different, and the morphology and porosity are the key factors affecting the mechanical properties of the sintered samples.

**Figure 5:**
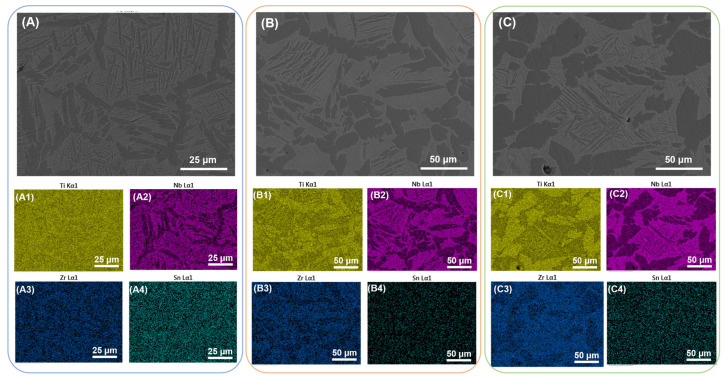
SEM images of the as-sintered samples and corresponding elemental maps: (**A**) and (**A1**–**A4**) S1400 sample; (**B**) and (**B1**–**B4**) S1450 sample; (**C**) and (**C1**–**C4**) S1500 sample.

The grain size of S1400 sample is about 60 μm, the spherical α phase is precipitated preferentially along the grain boundary, while the α plates are precipitated inside the grain. Raising the sintering temperature can enhance the driving force of microstructure growth, which makes the grain size of S1450 and S1500 sample grow up to 80 μm and 100 μm, and the α phase within the grain becomes coarse, correspondingly. With the elements partitioning during the sintering process, Nb and Zr are enriched in the β matrix and absent in the α phase, and neutral elements Sn distribute uniformly in the whole region without recognizable segregation [40], as shown in Figure 5.

### 3.3. Microstructure Characterization of Solution Treated Samples

**Figure 6:**
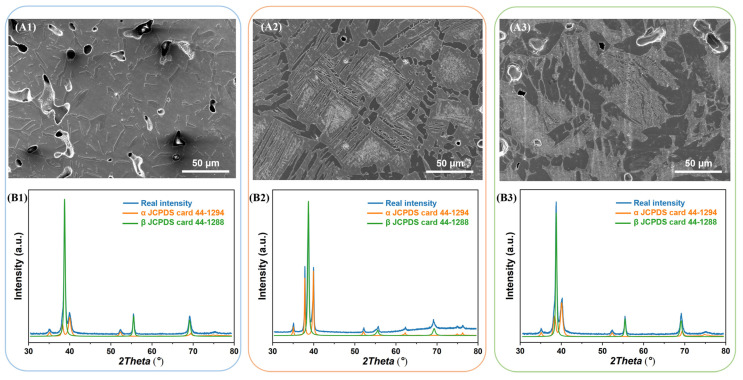
SEM images and XRD spectrums of the solution-treated samples: (**A1**,**B1**) S1400 + Q sample; (**A2**,**B2**) S1450 + Q sample; (**A3**,**B3**) S1500 + Q sample.

**Figure 7:**
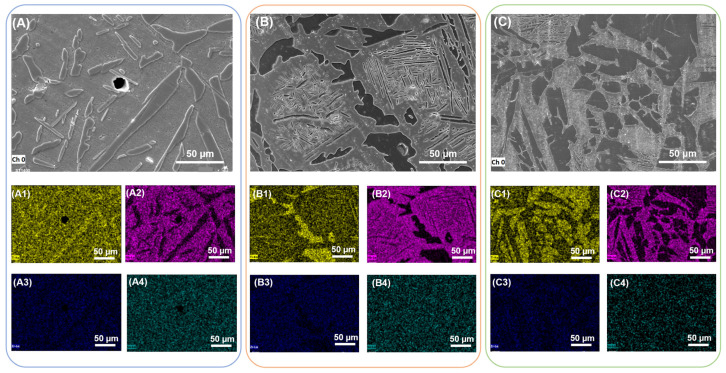
SEM images of the solution-treated samples and corresponding elemental maps: (**A**) and (**A1**–**A4**) S1400 + Q sample; (**B**) and (**B1**–**B4**) S1450 + Q sample; (**C**) and (**C1**–**C4**) S1500 + Q sample.

In order to further optimize the microstructure, reduce the porosity, and improve the comprehensive mechanical properties, we performed solution treatment on the samples prepared by the three sintering processes, which are abbreviated as S1400 + Q, S1450 + Q and S1500 + Q, respectively. As shown in Figure 6, the microstructure of the S1400 + Q sample has changed significantly, the acicular α phase inside the grain has disappeared, while the morphology of S1450 + Q and S1500 + Q was similar to that before the solution treatment. As shown in Figure 7, the results of EDS show that there is still obvious elemental separation in the sample. Based on the experimental results after solid solution treatment, water quenching did not achieve the ideal effect of inhibiting the α phase, especially the S1450 + Q and S1500 + Q samples, which may be related to the loss of β stability elements at high temperature sintering [41].

**Figure 8:**
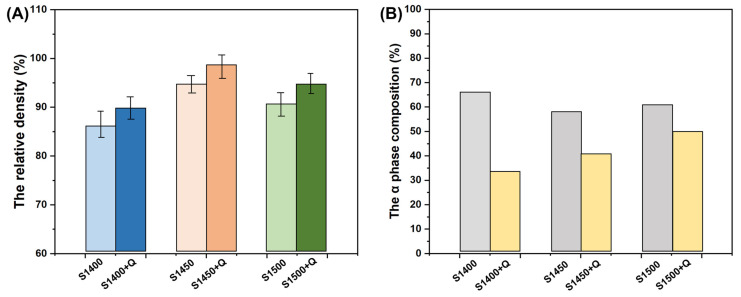
The relative density and phase composition of the as-sintered and water-quenched Ti2448 samples: (**A**) the relative density; (**B**) the α phase composition.

We employed the expectation maximization (EM) algorithm Rietveld refinement to fit the XRD patterns in order to estimate the volume fraction of the β and α phases. As shown in Figure 8, the α-precipitated phase fractions of the samples sintered at 1400 °C, 1450 °C, and 1500 °C were 66.7%, 58.5%, and 60.7%, respectively, while the densities were 87%, 95%, and 92%, respectively. Solid solution treatment increased the density of the sample, resulting in densities of 90%, 98%, and 95% for S1400 + Q, S1450 + Q, and S1500 + Q, respectively. In contrast, solid solution treatment decreased the volume fraction of the α-precipitated phase to 32.5%, 41.2%, and 46.9% for the same samples.

### 3.4. Mechanical Characterization and Fracture Analysis

**Figure 9:**
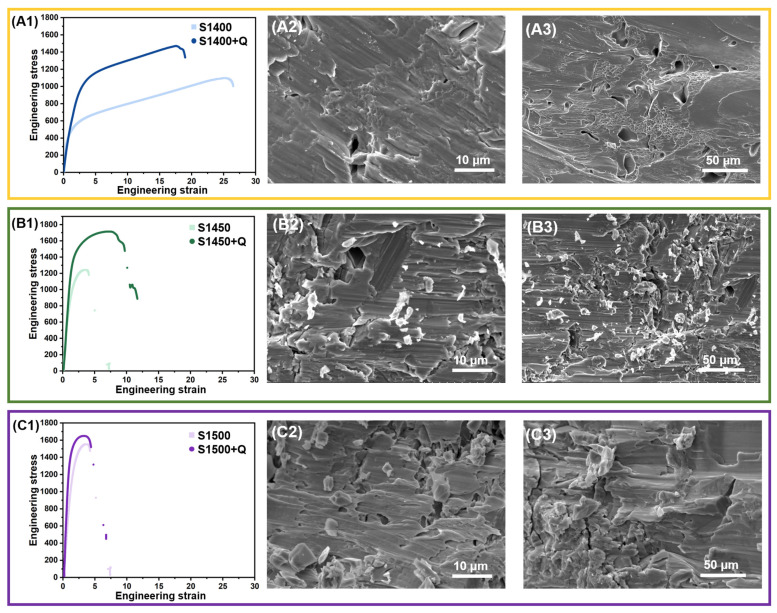
The compressive stress-strain curves and fracture topography of the as-sintered and water-quenched Ti2448 samples: (**A1**) the compressive stress-strain curves of S1400 and S1400 + Q sample; (**A2**) fracture topography of S1400 sample; (**A3**) fracture topography of S1400 + Q sample; (**B1**) the compressive stress-strain curves of S1450 and S1450 + Q sample; (**B2**) fracture topography of S1450 sample; (**B3**) fracture topography of S1450 + Q sample; (**C1**) the compressive stress-strain curves of S1500 and S1500 + Q sample; (**C2**) fracture topography of S1500 sample; (**C3**) fracture topography of S1500 + Q sample.

**Figure 10:**
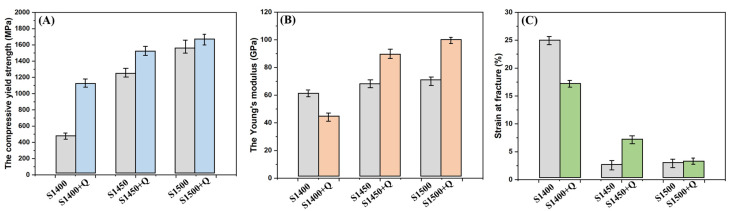
Mechanical properties of the as-sintered and water-quenched Ti2448 samples: (**A**) the compressive yield strength; (**B**) the Young’s modulus; (**C**) strain at fracture.

To characterize the mechanical properties, we performed uniaxial compression tests on the samples with different preparation processes, as shown in Figure 9. The compressive yield strength, the Young’s modulus, and the strain at fracture were extracted from the compressive stress-strain curves, and their values are shown in Figure 10. First of all, the sintering temperature has a significant influence on the mechanical properties of the specimen. The compressive yield strength of the S1400 sample is only 450 MPa, while the compressive yield strength of the S1450 and S1500 sample increases to 1250 MPa and 1450 MPa, respectively. On the contrary, the strain at fracture drops sharply with the elevation of the sintering temperature. The strain at fracture of the S1400 sample is 25%, while the strain at fracture of the S1450 and S1500 samples are only 3%. In addition, Young’s modulus is very important for the effectiveness of implants in vivo, and we strive to obtain samples with a modulus closer to that of human bone. As the sintering temperature elevates from 1400 °C to 1500 °C, the Young’s modulus rises from 62.5 GPa to 70.0 GPa. The presence of continuous GB-α may lead to a decrease in fracture-related performance, as it may obstruct the dislocation movement of adjacent α colonies and β matrix [42,43]. Dislocations accumulate in continuous GB-α, resulting in strain localization, which, in turn, promotes crack initiation [44,45].

Compared to the as-sintered samples, the solution treated samples exhibit much better mechanical properties, the compressive yield strength increased to 1100 MPa, 1550 MPa and 1650 MPa, respectively. The strain at fracture was relatively stable, dropped to 17.5% for the S1400 + Q sample, but slightly improved to 7.5% for the S1450 + Q sample, and was almost unchanged for the S1500 + Q sample. It benefitted from the substantial reduction of the α-phase after quenching and the high porosity, and the Young’s modulus of the S1400 + Q sample was further reduced to 44 GPa while the Young’s modulus of the S1450 + Q and S1500 + Q samples increased slightly due to the improvement of density. Furthermore, the fracture surfaces of the S1400 + Q sample has abundant dimples, which imply good ductility of the materials. However, dimples disappeared in the other samples and were replaced by cleavage fractures along the lamellar α-phase, as shown in Figure 9. The solution treatment can successfully suppress the precipitation of the α phase in the Ti2448 alloy, yielding samples with a bulk β phase [31]. Liu et al. made a detailed investigation into the deformation behavior of GB-α with different morphologies, and they concluded that discontinuous GB-α is considered beneficial to the fracture resistance, since the dislocation motion is hypothesized to easily transfer through prior-β grain boundaries at the sites without the GB-α [46,47]. Therefore, the water-quenched sample exhibits outstanding performance due to the absence of the acicular α-phase. The S1400 + Q sample benefits from high porosity and a finer microstructure, and its comprehensive mechanical properties are more suitable for orthopedic implant application.

## 4. Conclusions

We used high-purity Ti, Nb, Zr, and Sn powders as raw materials and mixed the powders at a mass ratio of 64:24:4:8 to obtain pre-alloyed Ti2448 powder via ball milling. We focused on the influence of the sintering process on the porosity, phase composition, and mechanical properties of the initial sintered state. Furthermore, we performed solution treatment on the samples under different sintering parameters to further modify the microstructure and phase composition in order to achieve strength enhancement and a reduction of Young’s modulus. Our work provides an economical and reliable process reference for promoting biomedical applications of Ti2448 alloys. The main conclusions are as follows:

(1) The as-sintered samples have a two-phase microstructure where the secondary α phase were inside the grains, while the primary α phase precipitated along the grain boundaries of the matrix β phase. Beyond elements partitioning during the sintering process, Nb is enriched in the β matrix and absent in the α phase.
(2) The proportions of the α-precipitated phases of the samples sintered at 1400 °C, 1450 °C, and 1500 °C were 66.7%, 58.5% and 60.7%, respectively, and the density was 87%, 95% and 92%, respectively.
(3) The microstructure of S1400 + Q sample changed significantly, the acicular α phase inside the grain disappeared, and the volume fraction of β phase was as high as 67.5%, while the morphology of S1450 + Q and S1500 + Q was similar to that before solution treatment, except that the porosity and the volume fraction of α phase reduced slightly.
(4) Solution treatment can effectively suppress the precipitation of α phase in Ti2448 alloy, yielding samples with a bulk β phase. Therefore, the water-quenched sample exhibits good mechanical properties due to the absence of continuous GB-α phase. The S1400 + Q sample benefits from high porosity and a finer microstructure, and it has excellent comprehensive mechanical properties (the compressive yield stress is 1100 MPa, the strain at fracture is 17.5% and the Young’s modulus is 44 GPa), which are more applicable to orthopedic implants.

## Data Availability

Not applicable.
